# Modeling Intestinal Epithelial Response to Interferon-γ in Induced Pluripotent Stem Cell-Derived Human Intestinal Organoids

**DOI:** 10.3390/ijms22010288

**Published:** 2020-12-30

**Authors:** Michael J. Workman, Elissa Troisi, Stephan R. Targan, Clive N. Svendsen, Robert J. Barrett

**Affiliations:** 1 Cedars-Sinai Medical Center, Board of Governors Regenerative Medicine Institute, Los Angeles, CA 90048, USA; Michael.Workman@cshs.org (M.J.W.); etroisi1@gmail.com (E.T.); Clive.Svendsen@cshs.org (C.N.S.); 2 Cedars-Sinai Medical Center, F. Widjaja Foundation Inflammatory Bowel and Immunobiology Research Institute, Los Angeles, CA 90048, USA; Stephan.Targan@cshs.org; 3 Cedars-Sinai Medical Center, Department of Biomedical Sciences, Los Angeles, CA 90048, USA

**Keywords:** iPSC-derived human intestinal organoids, disease modeling, IFN-γ

## Abstract

Human intestinal organoids (HIOs) are increasingly being used to model intestinal responses to various stimuli, yet few studies have confirmed the fidelity of this modeling system. Given that the interferon-gamma (IFN-γ) response has been well characterized in various other cell types, our goal was to characterize the response to IFN-γ in HIOs derived from induced pluripotent stem cells (iPSCs). To achieve this, iPSCs were directed to form HIOs and subsequently treated with IFN-γ. Our results demonstrate that IFN-γ phosphorylates STAT1 but has little effect on the expression or localization of tight and adherens junction proteins in HIOs. However, transcriptomic profiling by microarray revealed numerous upregulated genes such as *IDO1*, *GBP1*, *CXCL9*, *CXCL10* and *CXCL11*, which have previously been shown to be upregulated in other cell types in response to IFN-γ. Notably, “Response to Interferon Gamma” was determined to be one of the most significantly upregulated gene sets in IFN-γ-treated HIOs using gene set enrichment analysis. Interestingly, similar genes and pathways were upregulated in publicly available datasets contrasting the gene expression of in vivo biopsy tissue from patients with IBD against healthy controls. These data confirm that the iPSC-derived HIO modeling system represents an appropriate platform to evaluate the effects of various stimuli and specific environmental factors responsible for the alterations in the intestinal epithelium seen in various gastrointestinal conditions such as inflammatory bowel disease.

## 1. Introduction

The intestinal epithelium is a highly dynamic layer of columnar cells that physically separates the luminal contents of the intestine from the host and has numerous functions including nutrient absorption, regulation of the mucosal immune system, and control of intestinal permeability [1]. Given the pivotal role the intestinal epithelium plays, it is not surprising that dysfunction in this tissue is implicated in numerous intestinal conditions including inflammatory bowel disease (IBD) [2], celiac disease [3] and cystic fibrosis [4]. Human intestinal organoids (HIOs), which can be generated from either induced pluripotent stem cells (iPSCs) [5] or intestinal biopsies [6,7], represent one avenue in which intestinal epithelial responses to various stimuli can be modeled and characterized. This system is particularly suitable for assaying such responses given that organoids from both cellular sources can be propagated for prolonged periods under tightly controlled culturing conditions, thereby allowing a precise examination of how these stimuli affect the functioning of this tissue.

While both iPSC- and biopsy-derived HIOs are increasingly being used to model responses to various cytokines and microbes [8,9], the goal of this study was to thoroughly characterize the effect of the pro-inflammatory cytokine interferon-gamma (IFN-γ) on iPSC-HIOs. IFN-γ was chosen for this study as it is known to be upregulated in IBD [10], its signaling transduction via phosphorylation of STAT1 (p-STAT1) is well described [11], and genes upregulated in other IFN-γ treated intestinal epithelial models have been identified [12,13,14]. Here, we demonstrate that our iPSC-derived organoid system is biologically responsive to IFN-γ, and that short-term exposure causes phosphorylation of STAT1. More prolonged exposure to IFN-γ causes upregulation of known IFN-γ target genes and identification of other previously unreported genes that are regulated by IFN-γ in the intestinal epithelium. Further exploration of the most significantly upregulated gene, indoleamine 2, 3-dioxygenase (*IDO1*), shows this gene is quickly upregulated following IFN-γ exposure and remains highly elevated throughout the duration of IFN-γ treatment. Analysis of tissue sections reveal that IDO1 is highly expressed in IFN-γ treated organoids and in involved colonic regions of patients with active Crohn’s disease. These results indicate that iPSC-derived HIOs are a suitable model to study the intestinal epithelial inflammatory response and further validate the approach of using this system to examine the effects of various stimuli associated with gastrointestinal disorders such as IBD.

## 2. Results

### 2.1. Human Intestinal Organoids Are Responsive to IFN-γ

**Figure 1:**
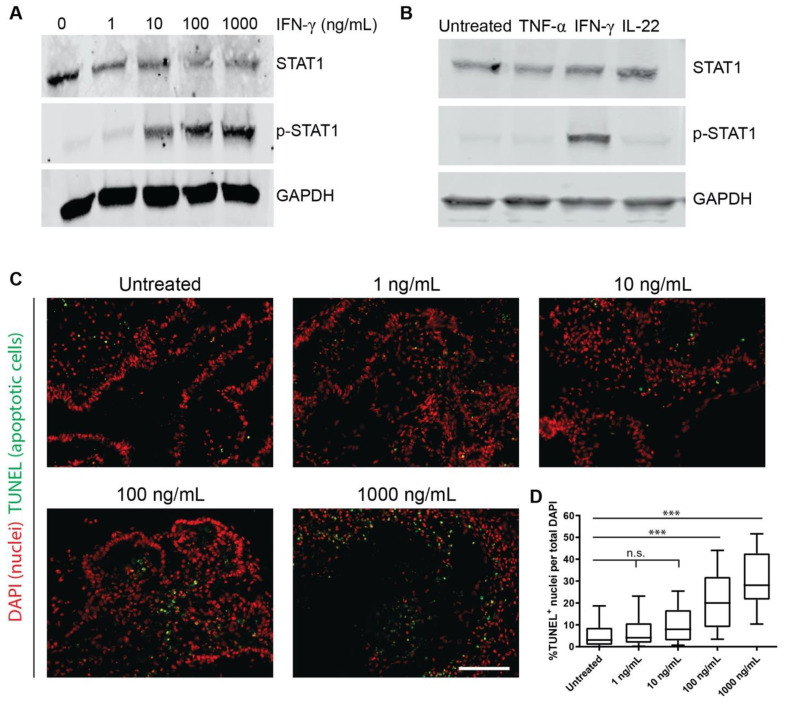
iPSC-derived intestinal organoids are responsive to interferon-gamma (IFN-γ). (**A**) Representative Western blot showing detection of STAT1, phosphorylated STAT1 (p-STAT1), and GAPDH loading control reveals that human intestinal organoids (HIOs) treated for 1 h with IFN-γ concentrations of 10 ng/mL and greater exhibit robust phosphorylation of STAT1. (**B**) Phosphorylation of STAT1 is specific to IFN-γ treatment. (**C**) Representative images of HIOs stained with TUNEL and DAPI. Scale Bar = 100 μm (**D**) Quantification of TUNEL+ nuclei in HIOs treated for 3 days with IFN-γ, showing an increase in apoptosis in HIOs treated with 100 ng/mL and 1000 ng/mL IFN-γ versus untreated controls. Plots represent the combined data from 3 independent HIO differentiations and IFN-γ treatments (*n* = 24 to 32 analyzed sections per treatment group). Boxes represent the 25th to 75th percentiles, whiskers represent the 10th to 90th percentiles, and the bar represents the median. *** *p* < 0.001, n.s.—not significant, ANOVA with Dunnett’s multiple comparisons test comparing each group to Untreated.

iPSCs from a control individual (CS83iCTR-n33) were cultured in mTeSR1 medium on Matrigel-coated plates under feeder-free conditions and induced to form three-dimensional HIOs, as previously described [15]. In order to establish that IFN-γ exerts a biological effect on HIOs, we first determined if incubation with IFN-γ resulted in the phosphorylation of STAT1, given that it is well-established that this cytokine mediates its canonical effects via this mechanism [11]. HIOs were incubated with various concentrations of IFN-γ ranging from 0 to 1000 ng/mL for 1 h. Minimal phosphorylated STAT1 (p-STAT1) was detected in HIOs treated with 0 and 1 ng/mL of IFN-γ; however, concentrations of 10–1000 ng/mL resulted in robust p-STAT1 (Figure 1A). We next tested the specificity of phosphorylation of STAT1 by IFN-γ by incubating organoids with two other cytokines with known roles in intestinal inflammation, TNF-α [16] and IL-22 [17]. Remarkably, these cytokines had no effect on the phosphorylation level of STAT1, which was confirmed to be an effect specific to IFN-γ treatment (Figure 1B).

HIOs were incubated under similar concentrations of IFN-γ, as described above, for a longer duration of three days to assess if such concentrations resulted in increased apoptosis. Terminal deoxynucleotidyl transferase dUTP nick-end labeling (TUNEL) revealed no significant increase in the number of apoptotic cells in HIOs treated with 1 ng/mL or 10 ng/mL of IFN-γ compared to untreated controls. However, HIOs incubated with 100 ng/mL and 1000 ng/mL exhibited a significant increase in TUNEL-positive cells (Figure 1C,D). Given that exposure of HIOs to 10 ng/mL of IFN-γ gave robust p-STAT1 yet did not result in significantly increased cell death, all future experiments were conducted with this concentration.

### 2.2. Acute IFN-γ Exposure Does Not Cause Overt Disruption to Junctional Complexes

**Figure 2:**
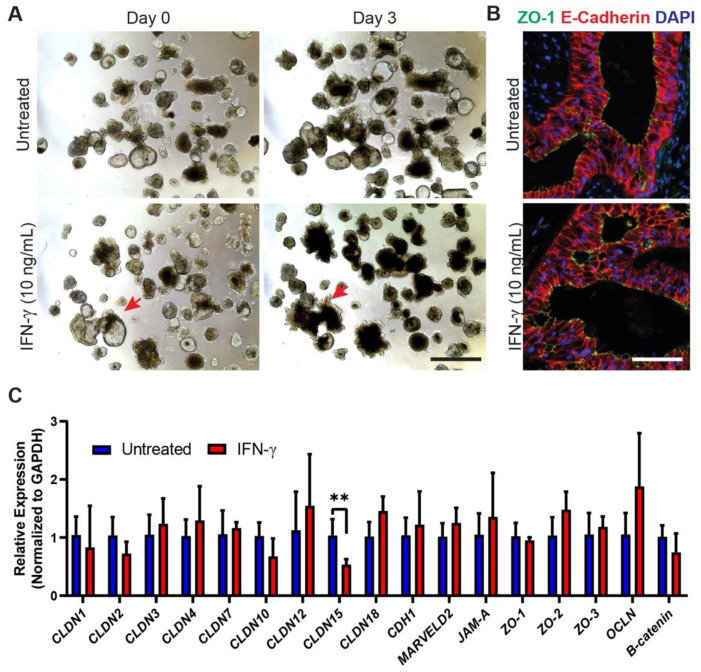
Acute IFN-γ exposure results in minimal disruption of junctional complexes in HIOs. (**A**) Gross morphology of HIOs after 3 days of IFN-γ treatment (10 ng/mL). IFN-γ-treated organoids displayed changes in morphology such as collapse of cystic-like organoid structures (red arrowheads). Scale Bar = 1 mm (**B**) Immunohistochemical staining of HIOs shows no disruption or mislocalization of ZO-1+ tight junctions or E-cadherin+ adherens junctions. Scale Bar = 100 μm (**C**) qPCR analysis of various junctional proteins shows minimal changes in gene expression levels in HIOs treated with IFN-γ (10 ng/mL). Notably, we detected a decrease in *CLDN15* expression. Data represent mean ± standard deviation with relative expression values being generated from 1–3 independent experiments (*n* = 3–9 biological replicates for each gene with each biological replicate value being averaged from 2 technical replicates of qPCR data). ** *p* < 0.01, multiple *t*-tests with Benjamini–Hochberg FDR correction.

HIOs were grown for 70 days under standard culture conditions and subsequently incubated with 10 ng/mL IFN-γ for 3 and 6 days. After exposure to IFN-γ for 3 days, organoids appeared smaller and darker (Figure 2A) with the collapse of some cystic-like organoids into more convoluted structures (Figure 2A, red arrowheads) that was not apparent in untreated controls. It has previously been reported that IFN-γ can affect intestinal permeability by altering the expression or eliciting the mislocalization of tight junctions and adherens junctions in intestinal epithelial cell lines [18,19]. Upon examination, we found no mislocalization of the tight junction protein ZO-1 or the adherens junction protein E-Cadherin in IFN-γ-treated HIOs, which appeared indistinguishable from untreated controls at the microscopic level using these markers (Figure 2B). We next analyzed by quantitative PCR (qPCR) the expression levels of a suite of genes encoding various junctional proteins to determine if any changes occurred at the transcriptional level in response to IFN-γ treatment. With the exception of a decrease in *CLDN15* expression, we found no significant difference in gene expression levels between IFN-γ-treated and untreated HIOs (Figure 2C).

### 2.3. Microarray Analysis Reveals a Significant Number of IFN-γ Regulated Genes in HIOs

**Figure 3:**
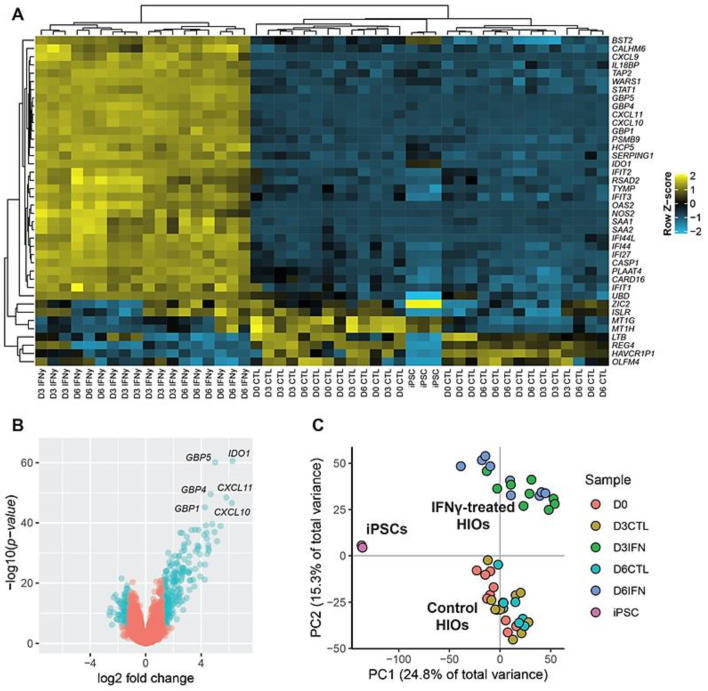
Analysis of microarray data from iPSCs and HIOs. (**A**) IFN-γ-treated HIOs completely segregate from all other samples upon unsupervised hierarchical clustering of the top 50 most highly variable genes determined by interquartile range. Data represent 3 independent experiments. (**B**) A volcano plot of differentially expressed genes shows that 326 genes are differentially expressed by a log2-fold change ≥ ±1.2 and adjusted *p*-value < 0.05 (blue circles). The most highly significant genes belong to the *IDO, CXCL,* and *GBP* classes of genes. (**C**) Principal component analysis of microarray expression data reveals a clear separation of iPSCs from HIOs along PC1 and a separation of IFN-γ-treated HIOs from untreated HIOs along PC2.

In order to more thoroughly characterize the effect of IFN-γ on HIOs, an expression microarray analysis was carried out. RNA was collected from iPSCs, untreated HIOs and IFN-γ-treated (10 ng/mL) HIOs at days 0, 3, and 6 and processed for hybridization on Illumina HumanHT-12 v4 BeadChips. After signal normalization and filtering for bad probes, analysis of the top 50 highly variable genes determined by interquartile range was used to cluster the samples. Remarkably, IFN-γ-treated HIOs completely segregated from all other samples using unsupervised hierarchical clustering (Figure 3A). Minimal differences between day 3 and day 6 IFN-γ-treated HIOs were observed.

We next performed differential gene expression analysis between untreated and treated HIOs to determine which genes are regulated by IFN-γ. Of the 41,181 probes detected on the Illumina HumanHT-12 v4 BeadChip, 16,113 met our filtering criteria. Of these, 4959 were determined to be differentially expressed between the control and IFN-γ treated groups (Benjamini–Hochberg false discovery rate adjusted *p* < 0.05) with 326 genes differentially expressed by a log_2_ fold change ≥ ±1.2 and adjusted *p*-value < 0.05 (Figure 3B). The majority of these genes were determined to be upregulated in IFN-γ-treated versus controls (237 upregulated, 89 downregulated). Principal component analysis (PCA) using all genes meeting the filtering criteria separated iPSCs from HIOs along principal component 1 (PC1), which represents 24.8% of the total variance in the data (Figure 3C). Separation along PC1 is largely driven by the enrichment of pluripotency genes in iPSCs such as *POU5F1, NANOG, LIN28A,* and *SOX2*, which are the top genes in the PC1-negative direction; and enrichment of intestinal genes such as *CDX2, OLFM4, KLF5, CEACAM, TFF*, and *MUC* genes which represent the top genes in the PC1-positive direction enriched in HIOs. PC2 separates IFN-γ-treated HIOs from untreated controls, with day 3 and day 6 HIOs being interspersed throughout each treatment cluster (Figure 3C), again suggesting minimal differences between day 3 and day 6 timepoints.

### 2.4. Gene Set Enrichment Analysis of IFN-γ Treated HIOs Reveals Pathways Associated With Immune Response

**Figure 4:**
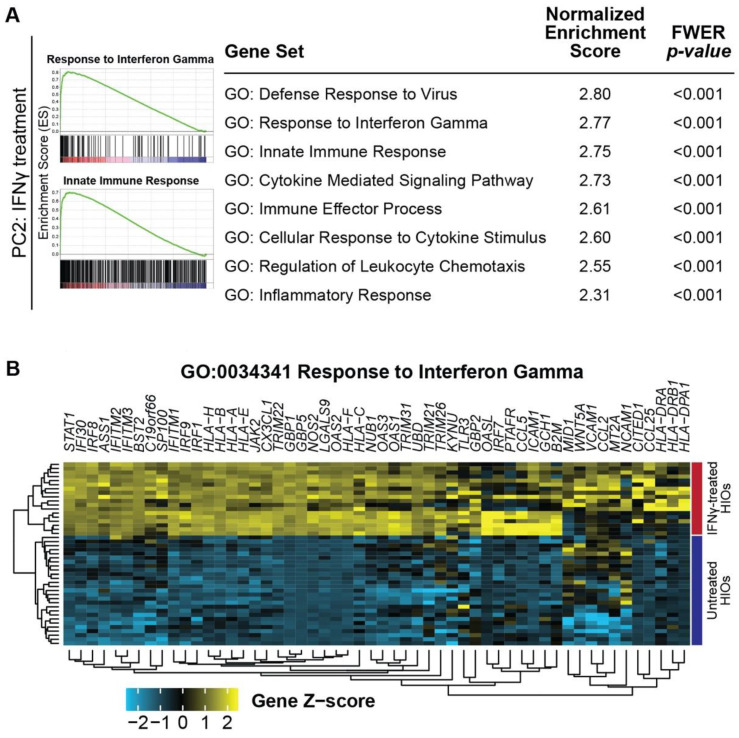
GSEA reveals enrichment of immune-related pathways in IFN-γ-treated HIOs. (**A**) Enrichment plots and enriched GO term gene sets in the ranked PC2 gene loadings using gene set enrichment analysis (GSEA). (**B**) Heatmap of the leading edge genes driving enrichment of ‘GO: Response to Interferon Gamma’ in IFN-γ-treated HIOs.

To gain insight into the many differentially expressed genes discovered using our microarray-based gene expression approach, we performed gene set enrichment analysis (GSEA). This approach focuses on finding enrichment of gene expression changes in pathways or gene sets that may not be easily apparent from single-gene analysis [20]. Using this method, we examined pathways associated with the separation of IFN-γ-treated and untreated HIOs along PC2 (Figure 3C). Using the ranked gene loadings from PC2, we performed GSEA against the complete set of gene ontology (GO) terms associated with biological process, molecular function, and cellular component. Pathways related to immune response and interferon signaling were enriched in the positive gene loadings along PC2, which are associated with IFN-γ-treated HIOs (Figure 4A). One of the most highly enriched gene sets was “GO: Response to Interferon Gamma”, with a normalized enrichment score of 2.77 and family-wise error rate (FWER) of *p* < 0.001, providing some validation of iPSC-derived HIOs as a suitable platform for modeling response to IFN-γ. Analysis of the leading edge genes responsible for the enrichment of “GO: Response to Interferon Gamma” revealed that these genes are highly upregulated in IFN-γ-treated HIOs and contain many known IFN targets and immune-related human leukocyte antigen (HLA) encoding genes (Figure 4B).

### 2.5. HIO Response to IFN-γ Is Independent of iPSC Line and HIO Age

**Figure 5:**
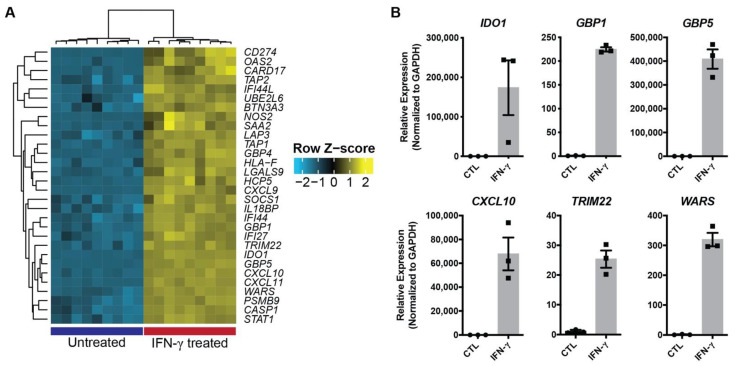
IFN-γ-induced gene expression changes are independent of cell line and age. (**A**) Heatmap of the top differentially expressed genes determined by microarray analysis in 70-day HIOs treated with IFN-γ and derived from iPSC line CS83iCTR-n33. (**B**) Gene expression changes of 6 selected IFN-γ-responsive genes determined by qPCR in 30-day HIOs differentiated from iPSC line (CS688iCTR-n5).

To confirm that the response to IFN-γ was not iPSC-line- or time-dependent, we examined the expression of the top genes regulated by IFN-γ in HIOs differentiated from a different iPSC line (CS688iCTR-n5) and analyzed at an earlier 30-day timepoint. Our previous analysis using the CS83iCTR-n33 line examined HIO response to IFN-γ at 70 days in culture and found by microarray that the most significant differentially expressed genes were all upregulated in IFN-γ-treated HIOs (Figure 5A). Using the same differentiation approach, we generated HIOs from the CS688iCTR-n5 iPSC line and treated with 10 ng/mL IFN-γ for 3 days starting on day 30. Similar to results with day 70 organoids generated from CS83iCTRn33, we found significant upregulation of the six confirmatory genes we chose to analyze by qPCR (Figure 5B). These results suggest the HIO response to IFN-γ is independent of the cell line used to generate the HIOs, and that HIOs are responsive at a much earlier timepoint of 30 days.

### 2.6. IFN-γ-Treated HIOs Model IBD Relevant Changes in Intestinal Epithelium

**Figure 6:**
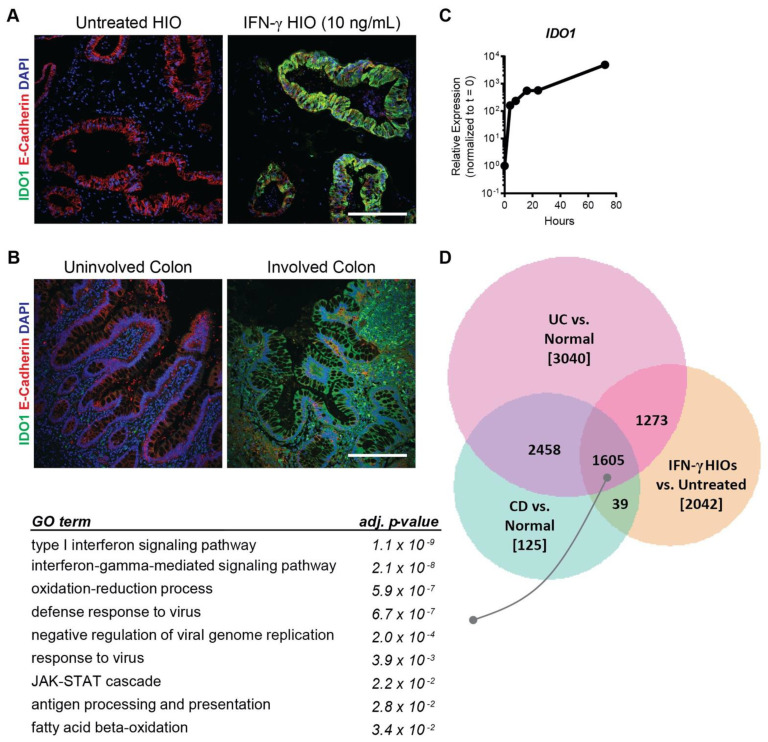
iPSC-derived organoids capture IBD-relevant changes in intestinal tissue. (**A**) IFN-γ-treated HIOs display significant upregulation of IDO1. Scale Bar = 200 μm (**B**) Colonic biopsies from Crohn’s disease patients show similar upregulation of IDO1 in involved segments of intestinal tissue. Scale Bar = 200 μm (**C**) *IDO1* is highly upregulated within 4 h of IFN-γ exposure and stays elevated throughout treatment. (**D**) Overlap of differentially expressed genes in IFN-γ-treated HIOs and CD and UC biopsies versus untreated HIOs and healthy control biopsies, respectively, shows enrichment of pathways associated with interferon signaling, immune response, and JAK-STAT cascade.

We next assessed tissue sections for protein expression of IDO1, the most highly upregulated gene in response to IFN-γ treatment discovered in our microarray analysis. IDO1 is an enzyme involved in tryptophan metabolism and has known roles in regulating T-cell proliferation and survival, as well as being involved in antimicrobial response [14]. IDO1 expression is known to be upregulated in several intestinal diseases, including IBD, where it has been shown to be highly expressed in inflamed colonic lesions of Crohn’s disease patients [21]. We therefore analyzed the expression of IDO1 in our IFN-γ-treated HIOs and found significant upregulation of IDO1 in both the epithelium and surrounding mesenchyme compared to untreated controls, where IDO1 was nearly undetectable (Figure 6A). An analysis of colonic biopsy sections from patients with Crohn’s disease revealed a similar upregulation of IDO1, specifically in involved regions (Figure 6B). A time course analysis of *IDO1* gene expression in HIOs revealed that this gene is significantly upregulated within 4 h of IFN-γ exposure and remains elevated throughout the duration of treatment (Figure 6C), indicating that *IDO1* is an early response gene to IFN-γ. Taken together, these results suggest that iPSC-derived HIO models can be used to study inflammatory processes associated with intestinal diseases such as IBD.

In order to understand the degree to which our IFN-γ-treated HIO model recapitulates aspects of Crohn’s disease (CD) and ulcerative colitis (UC), the two main forms of IBD, we compared our gene expression data against publicly available datasets, contrasting gene expression of in vivo biopsy tissue from patients with IBD against healthy control [22]. Comparison of differentially expressed genes between (1) IFN-γ-treated HIOs vs. untreated, (2) CD biopsies vs. healthy control biopsies, and (3) UC biopsies vs. healthy control biopsies revealed an overlap of 1605 genes between all three groups (Figure 6D). GO analysis of the overlapping genes revealed enrichment of pathways specific to processes such as interferon signaling, immune response, and JAK-STAT cascade (Figure 6D). These results demonstrate that iPSC-derived HIOs capture disease-relevant changes in intestinal tissue that correlate well with in vivo data collected from patient biopsies and represent an effective platform to investigate intestinal epithelial response to cytokines.

## 3. Discussion

HIOs are increasingly being used to model intestinal epithelial responses to various stimuli, and our goal was to validate this approach. To achieve this, we thoroughly characterized the IFN-γ response in iPSC-derived HIOs, and our findings provide numerous lines of evidence that support the utility of this modeling system. Firstly, as expected, administration of IFN-γ elicited the phosphorylation of STAT-1 (Figure 1A), confirming previous reports [11], while the lack of response to TNF-α and IL-22 (Figure 1B) confirmed the specificity of this response. Having determined that 10 ng/mL was the optimal concentration in which to culture HIOs with IFN-γ (Figure 1), we first examined the effect of this cytokine on tight and adherens junctions in HIOs. We report that there was no change in the localization of ZO-1 and E-cadherin, which is similar to a previous study whereby T84 cells were treated with IFN-γ [18], but conflicts with another which reported a substantial change in ZO-1 [19]. The reasons for these differences may be due to the fact that we are using a more physiologically relevant organoid system and that the conflicting study used 100 ng/mL, which was considerably higher than our concentration. While there may be conflicting data with regard to the sole addition of IFN-γ, numerous studies in adenocarcinoma lines have shown that the addition of IFN-γ with TNF-α causes mislocalization of ZO-1 and E-cadherin [23,24,25]. Although we did not examine the effect of co-administration of TNF-α and IFN-γ in this study, we did in a recently published report and found that the addition of both cytokines does cause mislocalization of ZO-1 and E-cadherin in iPSC-derived intestinal epithelium [26], and thus additionally supports our model.

Further validation of this modeling system is demonstrated from our microarray results, which show that HIOs are responsive to IFN-γ and a large number of genes are differentially expressed downstream of IFN-γ exposure (Figure 3A,B). In agreement with studies carried out in adenocarcinoma cell lines, we found similar upregulation of genes such as *IDO1* [14], *GBP1* [12], *CXCL9*, *CXCL10*, and *CXCL11* [13] in response to IFN-γ (Figure 3A), all of which are also known to be upregulated in IBD patients. Interestingly, genes encoding guanylate binding proteins (GBPs) were among the most significantly upregulated in the IFN-γ-treated group. GBPs have known roles in mammalian host defense, with GBP5 promoting inflammasome assembly [27] and GBP1 preventing intestinal epithelial cell apoptosis and regulating T-cell receptor signaling in the gut [28]. We also observed consistent upregulation of *CASP1* in IFN-γ-treated organoids, further supporting inflammasome assembly. However, given that we did not detect increased cell death with 10 ng/mL IFN-γ treatment, (Figure 1C,D) suggests that low doses of IFN-γ may lead to inflammasome activation without pyroptosis. Interestingly, adult intestinal stem cell markers such as *OLFM4* were significantly downregulated with IFN-γ treatment (Figure 3A) similar to the IFN-γ-mediated fetal reversion of adult intestinal crypts that has been observed in response to epithelial injury in mice (Nusse et al., Nature 2018). The finding that a subset of selected genes is similarly upregulated in IFN-γ-treated HIOs derived from another iPSC line and assayed at an alternative timepoint, demonstrates the broad applicability of this approach. Our finding that *IDO1* is upregulated in both colonic IBD samples and in our treated HIOs (Figure 6A,B) shows the utility of our model for recapitulating in vivo disease processes, given that IDO1 is known to be upregulated in IBD and shown to be highly expressed in inflamed colonic lesions of Crohn’s disease patients [21]. Finally, by overlapping the gene expression data generated in iPSC-derived HIOs with in vivo patient data, it was determined that HIOs can capture a large portion of gene expression changes observed in patients with active CD and UC, which are specifically enriched for interferon signaling and immune response (Figure 6D).

To conclude, this system will likely be useful for the examination of how specific environmental factors and other disease-relevant cytokines influence the functioning of the intestinal epithelium in a similar manner as described here for IFN-γ. Given that we can generate HIOs from iPSCs and have demonstrated we can reprogram lymphoblastoid cell lines (LCLs) to form iPSCs [29], this allows us and others to utilize biorepositories of LCLs to assess these responses in various genetic backgrounds. IBD is a complex polygenic disorder in which over 200 disease-associated risk loci have been discovered [30,31,32] and numerous clinical subtypes of disease exist [33]. Using iPSC-derived HIOs presented here or by incorporating them onto transwells [26] or into small microengineered Chips [15], we can begin to define personalized responses to various environmental factors and cytokines and model therapeutic outcomes in a precision medicine-based manner, which ultimately may improve drug selection for the treatment of IBD.

## 4. Materials and Methods

### 4.1. Cell Lines and Culturing

Karyotypically normal iPSC lines CS83iCTR-n33 and CS688iCTR-n5 were generated from healthy control individuals by the iPSC Core at Cedars-Sinai Medical Center. iPSCs were maintained under feeder free conditions on Matrigel matrix (Corning, Corning, NY, USA) in mTeSR1 media (Stem Cell Technologies, Vancouver, BC, Canada) and passaged every 4–5 days using StemPro EZPassage Tool (Thermo Fisher Scientific, Waltham, MA, USA). Cultures were determined to be negative for mycoplasma contamination using the MycoAlert Mycoplasma Detection Kit (Lonza, Basel, Switzerland).

### 4.2. HIO Generation from iPSCs

Human intestinal organoids (HIOs) were generated from iPSCs as previously described [15]. Briefly, iPSCs were passaged at approximately 80% confluency using a StemPro EZPassage Tool into 24-well tissue culture plates. Two days after passaging, mTeSR1 media were replaced with RPMI 1640 containing Activin A (100 ng/mL) and Wnt3A (25 ng/mL). On day 2 of differentiation, Wnt3A was removed and 0.2% and 2% FBS was added on days 2 and 3, respectively. Media were then replaced daily for 4 days with Advanced DMEM/F12 (Gibco) containing FBS (2%, *v*/*v*), FGF-4 (500 ng/mL), and CHIR99021 (3 µM, Tocris Bioscience, Minneapolis, MN, USA). Following differentiation, free floating and loosely attached intestinal tissue was embedded in Matrigel (Corning) and overlaid with Advanced DMEM/F12, B27 (1X), EGF (100 ng/mL), Noggin (100 ng/mL), CHIR99021 (2 µM, Tocris Bioscience), L-glutamine (5% *v*/*v*), and penicillin-streptomycin. All growth factors were from R&D systems unless otherwise noted.

### 4.3. TUNEL Staining in IFN-γ Treated HIOs to Detect Apoptotic Cells

Human intestinal organoids treated with varying concentrations of IFN-γ were processed and stained using the DeadEnd™ Fluorometric TUNEL System (Promega, Madison, WI, USA) following the manufacturer’s directions. Briefly, HIOs were washed with 1× PBS and fixed in 4% paraformaldehyde for 25 min at room temperature. Following fixation, HIOs were washed, embedded in OCT, and sectioned on a cryostat set at 10 µm. Sections were permeabilized in 0.25% Triton X-100 in 1× PBS for 5 min and incubated in equilibration buffer for 10 min at room temperature. Following equilibration, sections were stained with TdT reaction mixture for 60 min in the dark at 37 °C, washed in 1× PBS, and counterstained with DAPI to label nuclei. Slides were imaged on a Leica DM6000 B microscope and Image J software was used to quantify percent TUNEL+ nuclei per total nuclei.

### 4.4. Transcriptional Profiling of Intestinal Organoids by Microarray

HIOs were grown and passaged as described for approximately 70 days. Organoids were then treated with IFN-γ (10 ng/mL) in intestinal growth media and collected on days 3 and 6 along with untreated controls. HIOs were washed once with 1× PBS and lysed with 350 µL Buffer RLT Lysis Buffer (Qiagen, Hilden, Germany). RNA was isolated using the Qiagen RNeasy Mini Kit according to the manufacturer’s directions and transferred to the UCLA Technology Center for Genomics & Bioinformatics, where RNA was processed and hybridized to the Illumina HumanHT-12 v4 BeadChip. Raw data were normalized and processed using Limma in R. Our filtering criteria were as follows (1) removal of probes with ‘No match’ to gene symbols determined using illuminaHumanv4.db annotation data, (2) removal of probes annotated as ‘Bad’ with probe intensities greater than 12, and (3) filtering of probes not expressed in at least three arrays with a detection *p*-value < 0.05.

### 4.5. Quantitative Polymerase Chain Reaction (qPCR)

RNA was obtained from HIOs as described above. cDNA was generated from 1 µg of RNA using the SuperScript IV First-Strand Synthesis System (ThermoFisher Scientific, Waltham, MA, USA). Quantitative real-time polymerase chain reaction was performed using SYBR Select Master Mix (Applied Biosystems, Carlsbad, CA, USA) on a BioRad CFX384 Real-Time System. Forward and reverse primers were designed, when possible, to overlap exon–exon boundaries using the NCBI Primer-BLAST tool (https://www.ncbi.nlm.nih.gov/tools/primer-blast/) and used at a final concentration of 3 nM each (Table 1).

### 4.6. Western Blot Analysis

HIOs were washed with 1 PBS and total protein was extracted in situ using a cell lysis buffer supplemented with protease and phosphatase inhibitor (# MSSAFE, Sigma-Aldrich, Carlsbad, CA, USA). Protein concentration was then determined, and equal amounts of protein lysates were heat-denatured and separated on a 10% mini-protean precast gel (Bio-Rad, Hercules, CA, USA). Gel was then transferred on to midi format 0.2 µm Nitrocellulose membrane (Bio-Rad). Blot was blocked in Odyssey Blocking Buffer (LI-COR, Lincoln, NE, USA) for 1 h and then incubated in primary antibody (Table 2) for STAT1, pSTAT1, and glyceraldehyde-3-phosphate dehydrogenase (GAPDH) overnight at 4 °C. Blots were washed three times for 10 min each in 1× tris-buffered saline + 0.1% Tween-20 (TBST). After incubation with infrared conjugated secondary antibodies (LI-COR Biosciences, Lincoln, NE, USA) for 1 h, blots were washed with TBST and bands were visualized by scanning membrane on Odyssey Infrared Imaging System (Li-COR Biosciences).

### 4.7. Immunocytochemistry and Microscopy

HIOs were washed with 1× PBS, fixed in 4% PFA, transferred to 30% sucrose in 1× PBS (overnight at 4 °C), embedded in Tissue-Tek O.C.T compound (VWR, Radnor, PA, USA), and cut into 10 µm sections. Sections were then blocked in 10% normal donkey serum (Jackson ImmunoResearch, West Grove, PA, USA) and incubated with primary antibodies (Table 2) for either 3 h at room temperature or overnight at 4 °C. Sections were then rinsed and incubated in species-specific Alexa Fluor 488, Alexa Fluor 594, or Alexa Fluor 647-conjugated secondary antibodies (Life Technologies, Carlsbad, CA, USA) for 1 h at room temperature followed by DAPI (0.5 mg/mL; Life Technologies) for 10 min to counterstain nuclei. Chips and slides were imaged using a Leica DM6000 B microscope, or a Leica AF3500 microscope (Wetzlar, Germany).

### 4.8. Antibodies Used for Immunocytochemistry and Western Blot Analysis

## Figures and Tables

**Table 1:** Primer sequences used for qPCR.

Gene Name	Forward Primer	Reverse Primer
*GAPDH*	CTCTGCTCCTCCTGTTCGAC	TTAAAAGCAGCCCTGGTGAC
*IDO1*	ACACTTTGCTAAAGGCGCTG	TGCCTTTCCAGCCAGACAAA
*GBP1*	AAACTTCAGGAACAGGAGCAAC	GGTACATGCCTTTCGTCGTCT
*GBP5*	CCCAACTTGAAACACTGCCTG	GCACCAGGTTCTTTAGACGAGA
*WARS*	CGACTGCATTGGGAAGATCAG	ATGGCACATGGGATAAGGCAC
*CXCL10*	TGGCATTCAAGGAGTACCTCTC	CGTGGACAAAATTGGCTTGC
*TRIM22*	ACTACTGGGTGGACGTGATG	GCCGAAGACACCAAAAGCAG

**Table 2:** Primary antibodies for immunocytochemistry and Western blot.

Antigen	Host Species	Manufacturer	Catalog Number
E-Cadherin	Goat	R&D Systems	AF648
GAPDH	Rabbit	Santa Cruz	sc-25778
IDO1	Rabbit	Novus Biologicals	NBP1-87703
p-Stat1(Y701)	Rabbit	Cell Signaling	9167S
Stat1	Mouse	Cell Signaling	9176S
ZO-1	Mouse	Invitrogen	33-9100

## Data Availability

Data available upon request from the corresponding author.

## Abbreviations

HIO	human intestinal organoids
IDO1	Indoleamine 2,3-Dioxygenase 1
iPSC	induced pluripotent stem cell
IBD	Inflammatory bowel disease
IFN-γ	interferon-gamma
p-STAT1	phosphorylated STAT1
PC	principal component
